# CHAMP: Cognitive behaviour therapy for health anxiety in medical patients, a randomised controlled trial

**DOI:** 10.1186/1471-244X-11-99

**Published:** 2011-06-14

**Authors:** Peter Tyrer, Sylvia Cooper, Helen Tyrer, Paul Salkovskis, Mike Crawford, John Green, Georgina Smith, Steven Reid, Simon Dupont, David Murphy, Sarah Byford, Duolao Wang, Barbara Barrett

**Affiliations:** 1 Centre for Mental Health, Imperial College, Claybrook Road London, W6 8LN, UK; 2 Department of Psychology, University of Bath, Bath, BA2 7AY, UK; 3 Central and North West London NHS Foundation Trust, Hampstead Road, London, NW1 7QY, UK; 4 Greenacres Centre, Hillingdon Hospital, Pield Heath Road, Uxbridge UB8 3NN, UK; 5 Department of Clinical Psychology, 10th Floor - West Wing, Charing Cross Hospital, Fulham Palace Road, London, W6 8RF, UK; 6 Centre for the Economics of Mental Health, King's College London, De Crespigny Park, London SE5 8AF, UK; 7 Department of Medical Statistics, London School of Hygiene and Tropical Medicine, London WC1E 7HT, UK

## Abstract

**Background:**

Abnormal health anxiety, also called hypochondriasis, has been successfully treated by cognitive behaviour therapy (CBT) in patients recruited from primary care, but only one pilot trial has been carried out among those attending secondary medical clinics where health anxiety is likely to be more common and have a greater impact on services. The CHAMP study extends this work to examine both the clinical and cost effectiveness of CBT in this population.

**Method/Design:**

The study is a randomized controlled trial with two parallel arms and equal randomization of 466 eligible patients (assuming a 20% drop-out) to an active treatment group of 5-10 sessions of cognitive behaviour therapy and to a control group. The aim at baseline, after completion of all assessments but before randomization, was to give a standard simple explanation of the nature of health anxiety for all participants. Subsequently the control group was to receive whatever care might usually be available in the clinics, which is normally a combination of clinical assessment, appropriate tests and reassurance. Those allocated to the active treatment group were planned to receive between 5 and 10 sessions of an adapted form of cognitive behaviour therapy based on the Salkovskis/Warwick model, in which a set of treatment strategies are chosen aimed at helping patients understand the factors that drive and maintain health anxiety. The therapy was planned to be given by graduate research workers, nurses or other health professionals trained for this intervention whom would also have their competence assessed independently during the course of treatment. The primary outcome is reduction in health anxiety symptoms after one year and the main secondary outcome is the cost of care after two years.

**Discussion:**

This represents the first trial of adapted cognitive behaviour therapy in health anxiety that is large enough to test not only the clinical benefits of treatment but also whether the cost of treatment is offset by savings from reduced use of other health services in comparison to the control group.

Cognitive behaviour therapy for Health Anxiety in Medical Patients (CHAMP)

**Trial registration:**

Current Controlled Trials ISRCTN14565822

## Background

Abnormal health anxiety - and its older synonym, hypochondriasis - is a relatively common problem in both primary and secondary medical care settings [1-3]. It also places a substantial burden on health services [4], as its central feature is sufficient fear of having a serious disease to lead to medical consultation, and, very commonly, this is followed by further investigations. This condition, despite its ubiquity and its ability to provoke considerable suffering, is often unrecognised or appreciated at a superficial level. Even when the condition is recognised, concerns over litigation may lead to expensive investigations being carried out unnecessarily. This may provoke further pathology when findings of marginal clinical significance are reported. The extra burden on services is particularly important in secondary medical care. Between 10 and 20% of all attenders at medical clinics have abnormal health anxiety [5-7] and patients often rotate between different clinics depending on the focus of their symptoms. The symptoms of abnormal health anxiety show little tendency to spontaneous resolution and persist for months in the absence of treatment [5].

The failure to detect this serious pathology is perhaps less surprising when there is a general belief that there is no adequate treatment. Pharmacological management is generally unsatisfactory but psychological treatment in the form of cognitive behaviour therapy (CBT) has been shown to be effective, mainly in primary care [8,9] and, more recently, in a secondary care medical clinic [10]. Although these trials suggest efficacy of this intervention, it is less clear if it has a significant impact on costs. Although the total costs were somewhat reduced in those receiving cognitive behaviour therapy in the only trial in secondary care, these only became manifest in the six months after treatment had been completed [10]. The CHAMP trial is the natural successor to these studies, and examines both efficacy and cost-effectiveness of this newly modified treatment over a two-year period.

## Research objectives

The main objectives of the CHAMP trial are to examine the clinical value, cost and cost-effectiveness of the administration of CBT in health anxious patients attending out-patient clinics in secondary care. Specifically we hypothesised that (i) between 5 and 10 sessions of health anxiety directed cognitive behaviour therapy using the Salkovskis/Warwick model [11,12] would be more effective than normal care in the clinic in reducing abnormal health anxiety recorded by changes in the Health Anxiety Inventory (HAI)[13] one year after randomisation to the trial, and (ii) the cost of the CBT and control are equivalent, the endpoint for this outcome is the cost of health service interventions at 2 years adjusted for baseline values.

The secondary hypotheses are that, compared with the control condition, health anxiety directed cognitive behaviour therapy will lead to significantly greater improvement in health anxiety at 3, 6 and 24 months measured using the Health Anxiety Inventory (HAI)[13], in self-rated generalized anxiety measured using the Anxiety section of the Hospital Anxiety and Depression Rating Scale (HADS-A)[14], in quality of life using the EQ-5D measure of health-related quality of life [15], and in social functioning using the Social Functioning Questionnaire (SFQ)[16], all at 6, 12 and 24 months, result in the loss of the diagnosis of hypochondriasis at 24 months using the criteria of the Structured Clinical Interview for DSM-IV [17] and will be a cost-effective use of resources, where improvements in outcomes (measured using the HAI and in QALYs) are considered worthwhile.

In addition we will test the hypotheses that health anxiety focused cognitive behaviour therapy will be less effective in patients who have additional comorbid pathology in the form of (i) obsessional symptomatology (using the Short Obsessive Compulsive Screening (SOCS)[18]), dependent personality (using the Dependent Personality Questionnaire [19]), and hypochondriacal and other personality disorders [20] (using the Personality Assessment Schedule-Quick Version (PAS-Q)[21]) and that these comorbid disorders will be associated with increased costs.

The study also allows prevalence estimates to be made for health anxiety in different age groups and in different medical clinics.

## Methods/Design

### Trial summary

The study proposed is a pragmatic randomized controlled trial with two parallel arms and approximately equal randomization of eligible patients to an active treatment group of 5-10 sessions of cognitive behaviour therapy or to a control group. During the course of baseline assessment an explanatory interview will be given about the nature of health anxiety; this will be the only specific intervention in the control group but there is some evidence that this is of benefit in its own right [10].

### Settings

Patients attending cardiology, endocrine, gastroenterology, neurology and respiratory medicine clinics in five general hospitals (Kings Mill, North Nottinghamshire; St Mary's Hospital, Paddington, London; Charing Cross Hospital, Fulham, London; Chelsea and Westminster Hospital, London; and The Hillingdon Hospital, Middlesex) were considered for the study. 107 consultants agreed to collaborate with the study and to allow their patients to be approached provided they were not considered inappropriate for the study on account of the severity of their physical pathology.

### Form of randomisation

After baseline assessment randomisation was carried out by a computerised system (Open Clinical Data Management System (Open-CDMS)) using block randomisation with no stratification in randomised blocks of four and six. The allocation was carried out by the UK Mental Health Research Network and was independent of CHAMP personnel.

### Target population and procedure

Patients attending clinics of the collaborating consultants, apart from those who have been specifically excluded (see below), were approached while waiting for their out-patient appointments and given the short form of the Health Anxiety Inventory (HAI)[13], a rating scale of 14 questions that takes 5-10 minutes to complete. Those that scored at least 20 on the scale were offered the opportunity to take part in the trial and given an information sheet about the study. Within a week of receiving the information sheet and agreeing to be contacted the researcher administered part of the Structured Clinical Interview for DSM-IV with questions covering the diagnosis of hypochondriasis, and if the patient satisfied the criteria for this diagnosis he or she was invited to give formal consent and complete baseline assessments. At this appointment all patients received a standard explanation of the nature and significance of health anxiety that put their problem into context, and this was followed by the assessments described below. After completion of the assessments the research assistant entered and registered each patient to an on-line system (OpenCDMS Clinical Data Management System) and this was automatically followed by the appropriate randomisation. The study coordinator was then informed of the details of the treatment arm allocation and passed on the details to the next available therapist. Equal allocation was made to either (i) active treatment group - between 5 and 10 60-minute sessions of cognitive behaviour therapy from a graduate researcher, or trained research nurse or equivalent professional, at the clinic backed up by three booklets specially written for patients, or (ii) a control group of normal care who would not receive specific treatment but had already received a short account of the essentials of health anxiety at baseline interview.

The patients were recruited over a 21 month period beginning in October 2008 and each will be followed up for two years. In addition to research assistants who were employed on the trial, help in recruitment was also provided by Clinical Studies Officers of the North London and East Midlands hubs of the Mental Health Research Network.

### Ethics

The study was approved by the Nottingham Research Ethics Committee 1 (08/H0403/56) to run on all the planned sites covered by the clinics.

### Inclusion and exclusion criteria

Patients suitable for inclusion were those who satisfied the criteria for excessive health anxiety above and were (i) aged between 16 and 75, (ii) permanently resident in the area, (iii) had sufficient understanding of English to read and complete the questionnaires, and (iv) had given written consent for the interviews, audio-taping of 50% of treatment sessions, and for access to their medical records. The presence of existing medical pathology, provided it was not new and requiring further investigation, was not a bar to treatment in the study. This decision was made as in an earlier study we found that many patients with existing pathology who also had high health anxiety benefited from the intervention [10].

Some of those who would otherwise satisfy the inclusion criteria above were excluded if (i) they were felt by their consultants to have a level of continuing major pathology that was too severe for them to take part in the study, (ii) they were currently being actively investigated for significant pathology suspected by the clinician and for whom cognitive behaviour therapy might confuse or cause distress, (iii) they had significant cognitive impairment, and (iv) they were currently under psychiatric care.

## Assessments

The following assessments were carried out at base-line only;

(i) personality assessment using the quick version of the Personality Assessment Schedule (PAS-Q)[21] but also including the questions from the hypochondriasis subsection of the full schedule [20],

(ii) the Short Obsessional Compulsive disorder Screener (SOCS)[18] (a set of 7 questions that identifies the likely presence of obsessive compulsive disorder, and

(iii) the Dependent Personality Questionnaire [18], an assessment of dependence traits. These are included as both dependent personality and obsessional symptoms associate with another condition may handicap or complicate treatment.

The remaining clinical assessments, in addition to the main health anxiety one (HAI) examined the presence of generalised anxiety and depression (measured with the HADS scale [14]) as these are common in patients with hypochondriasis, and also adversely affects quality of life (EQ-5D)[15], and social functioning (SFQ)[16]. These assessments were given at baseline and at 6m, 12m and 24m (or earlier and at intervening periods if drop-out or loss to follow-up was likely). To ensure the highest possible follow-up the intention was to contact patients after 18 months to be reminded of the 2 year assessment, and if for any reason (eg being out of the country), the final assessment might be made earlier. The HAI was also given at 3 months by post or by telephone with a research assistant. Service use data for the economic evaluation was collected using the Adult Service Use Schedule (AD-SUS), an interviewer-assessed instrument designed by one of the applicants and based on previous economic evaluations in adult mental health populations [22,23], and also by examination of computerised hospital records. AD-SUS data were recorded at baseline and at 6, 12 and 24 months. Where the AD-SUS data conflict with the data obtained from the computerised records the AD-SUS data will take precedence only when it refers to a contact at a hospital not included in the electronic data search, as the details of admission and investigations is more likely to be correct from official records than from personal retrospective recall. Data on workplace absenteeism and presenteeism [24] were also recorded at 6, 12 and 24 months follow-up. In addition the SCID-I hypochondriasis questions [17] are to be administered again at 24 months to determine if each patient still satisfies the requirements for DSM-IV hypochondriasis. All these instruments are given at face to face interview but also have the advantage that they can be given over the telephone if necessary, and this can be useful in reducing loss to follow up. All assessments are carried out by independent research assistants who have had no contact with the patient at other times and who are carefully masked to avoid disclosure of allocation (see below).

## Methods of overcoming bias

The independent central computerized system involved in randomization ensures no bias in allocation of treatments. As this is a single blind study there is always the danger of disclosure of the form of treatment by the patient, therapist or other investigators in the study. This is minimized by (a) patients being asked by research assessors not to disclose their treatment to the research assessors, (b) the assessors and therapists working in different areas, (c) ensuring that all follow-up interviews are conducted by a researcher is masked to the patient's allocation status. The procedure was that whenever a researcher was unblinded a different research colleague was asked to conduct all further follow-up interviews.

## Study interventions

### Cognitive behaviour therapy treatment arm

The aim is to replicate the conditions of treatment that would be likely to prevail in the future if the trial found benefit from CBT, as much as possible the planned cognitive behaviour therapy was given within or close to the clinic close to the clinic as patients are more likely to consider it to be an unexceptional aspect of their care, thus avoiding the potential for stigma associated with a referral to mental health services. At each centre we therefore trained a graduate research worker, research nurse or equivalent health professional to administer the treatment. Each patient was invited to receive between 5 and 10 sessions of cognitive behaviour therapy with additional adaptations for health anxiety developed by Helen Tyrer and Paul Salkovskis, which is reinforced by three booklets to be handed to patients at treatment sessions (Table 1). Each therapist received training from the senior practitioners in the study before taking on the care of patients.

**Table 1:** Essentials of specific elements of cognitive behaviour therapy for health anxiety

Listing of all symptoms and how the patient interprets them
Formulation of worst fears

Introducing possibility of alternative explanations (eg pie charts and pyramids)

Evaluation of risk of disease

Considering the price paid for health anxiety

Exploring the hypothesis of fear of disease being more likely than actual disease

Building evidence by identifying symptom patterns that are related to those of anxiety (ie diary keeping)

Reducing behaviour that maintains health anxiety such as excessive bodily checking and reassurance seeking

Because of the need for training, recruitment was planned to be slower in the first three months of the trial compared with the later phases of the study. Following this initial period, therapists had fortnightly supervision until the last patients had been recruited and treated. Therapists were also given three booklets of cognitive behaviour therapy developed for health anxiety prepared for training during treatment.

## Training and Fidelity of Intervention

Four of the authors (PS, GS, DM and HT) were involved in the training of therapists. These four therapists, together with Hilary Warwick (one of the originators of the treatment), are also involved in assessing treatment fidelity, by listening to a selection of taped sessions from each therapist.

Approximately half of all treatment sessions will be recorded and tapes or discs of these sessions given to patients to help in their progress. Fidelity will be tested using a health anxiety modification of the Cognitive Therapy Rating Scale [25] (the Health Anxiety Version; CTRS-HAV. The issue is important as better treatment fidelity with cognitive behaviour therapy has been associated with greater treatment effects [26,27].

## Sample size

We calculated sample sizes for both the primary outcome measure and the first secondary outcome measure, choosing the larger of the two for the study.

Based on the pilot study in a genitourinary medicine clinic [10], we assume that the true difference in the change of HAI between CBT and control at 1 year is 5.00 points and that the standard deviation for the change of HAI at 1 year is 7.58 points. With the above assumptions, the study will need 122 patients to detect the above difference with 95% power at a two sided 5% significance level. Taking into account an estimated drop-out rate of 20% drop-out, the sample size is therefore estimated to be 152 patients.

There remains no agreed approach to calculate the sample size required for an economic evaluation, particularly in areas such as health anxiety in which the willingness to pay for improvements in outcomes is unknown. Based on the pilot study, we considered that the CBT intervention would be cost-effective if it improved HAI score and was no more costly than the control treatment. The sample size calculation for the economic evaluation was therefore based on the total costs over 24 months being equivalent.

With a sample size of 186 per group, the study will have 80% power to reject the null hypothesis that the cost of the CBT and control are not equivalent (where the difference in mean costs is +/- £150) in favour of the alternative hypothesis that the means of the two groups are equivalent, assuming the expected difference in means is £0 and the common standard deviation is £580 (from pilot data).

The sample size is thus powered by the first secondary outcome of the CHAMP study, estimated as 466 patients, assuming a 20% drop-out by 24 months, leading to an estimated total of 372 completed with equal randomisation between groups. Thus with 466 patients, or fewer if the drop-out rate is less, the study is adequately powered to both detect the assumed difference in the primary outcome and assess the equivalence in the secondary economic outcome between CBT and control group. The analysis will close on the date on which the last patient has completed the two year follow-up.

## Statistical analysis

All primary statistical analysis will use the intention-to-treat principle. The primary endpoint will be analysed using a mixed model with time, treatment, time × treatment interaction as fixed effects, baseline measurement as covariate, and patient as random effect. The treatment difference at 12 months together with its 95% confidence interval will be derived from the mixed model.

Missing data will be treated as missing at random in the above mixed model analysis and no imputation will be made. To assess the sensitivity of the result to this assumption, the last observation carried forward (LOCF) strategy will be used to compute the missing HAI at during the follow up visits. Other assessments will be analysed in a similar way.

The primary economic evaluation will only include those for whom complete data at baseline, 12 and 24 months follow-up are available. Sensitivity analysis will then include those cases with missing data, where LOCF will be used to account for missing data. The following baseline characteristics of study participants with available and unavailable service use data will be compared: centre, gender, living arrangements, ethnicity, marital status, education, EQ-5D.

For each piece of service use information collected with the AD-SUS, a unit cost will be applied and the total costs calculated. The total cost per participant is calculated by summing all costs. All unit costs will be for the financial year 2008-2009. Costs in the second year will be discounted at a rate of 3.5%, as recommended by the National Institute for Health and Clinical Excellence [28].

The difference in mean costs between active treatment and control groups together with the 95% confidence interval will be derived. The equivalence margin is pre-specified as £150. The equivalence will be declared between active and control groups in terms of secondary economic outcome if the 95% confidence interval falls within (-£150, +£150).

Even in situations where equivalence or non-inferiority are demonstrated, exploration of the joint distribution of costs and effects in a cost-effectiveness analysis (CEA) is recommended to represent uncertainty [29] and to help interpret the economic results. For these reasons, we propose to undertake a CEA irrespective of whether or not non-inferiority in the primary clinical outcome is demonstrated. Cost-effectiveness will be assessed using the net benefit approach [30] with reference to Bosmans' methods (31) for economic evaluations alongside equivalence or non-inferiority trials.

The cost-effectiveness analysis will use the primary outcome measure the HAI and a cost-utility analysis will be completed using QALYs derived from the EQ-5D data. A number of one-way sensitivity analyses will test the robustness of the results to the assumptions made in the economic evaluation.

The numbers (with percentages) of losses to follow-up at 12, and 24 months after randomisation will be reported and compared between the treatment arms with absolute risk differences (95% Confidence Intervals). Deaths will be reported separately for each group. The CONSORT procedure will be used for reporting flow through the trial.

## Discussion

The CHAMP trial is the first large scale trial of cognitive behaviour therapy for health anxiety in secondary care. Although there is increasing evidence of the clinical benefits of this treatment in primary care [8,32,33] the added value of this treatment in secondary care in terms of cost-effectiveness is still uncertain, and explains the importance of the two year follow-up. This extends far beyond the time of treatment and will allow for the assessment of longer term cost savings as well as the maintenance of clinical benefit. Preliminary evidence from a study carried out in parallel with CHAMP, suggests that hospital cost savings are substantial when treatment is successful [34].

## Current status of trial

The trial assessed 28,991 patients during the 21 months of recruitment and 444 were randomised. Drop-out in the early stages of follow-up is less than 10%.

## Competing interests

The authors declare that they have no competing interests.

## Authors' contributions

The trial was initiated by PT and HT, who, with PS, MC, BB, SB, DM, SD, JG and SR, designed the structure of the trial. DW and BB were primarily involved in developing the statistical analysis plan. SC was the trial coordinator and organiser of the recruitment strategy. Aaron Beck, MD, acted as trial adviser. All authors read and approved the final manuscript.

## Pre-publication history

The pre-publication history for this paper can be accessed here:

http://www.biomedcentral.com/1471-244X/11/99/prepub
